# Patient‐Controlled Sedation in Port Implantation (PACSPI 2)—A Randomised Clinical Trial

**DOI:** 10.1111/aas.70148

**Published:** 2025-11-10

**Authors:** Stefanie Seifert, Knut Taxbro, Andreas Nilsson, Josip Azman, Michelle S. Chew, Fredrik Hammarskjöld

**Affiliations:** ^1^ Department of Anaesthesia and Intensive Care Medicine Ryhov County Hospital Jönköping Sweden; ^2^ Department of Biomedical and Clinical Sciences Linköping University Linköping Sweden; ^3^ Department of Anaesthesia and Intensive Care Linköping University Hospital Linköping Sweden; ^4^ Department of Medical and Health Sciences Linköping University Linköping Sweden; ^5^ Pain and Rehabilitation Department Linköping University Hospital Linköping Sweden; ^6^ Department of Perioperative Medicine and Intensive Care Karolinska University Hospital Stockholm Sweden

**Keywords:** analgosedation, central venous catheter insertion, pain score, patient‐controlled sedation, PCS, subcutaneous venous port implantation, TIVAD

## Abstract

Optimising pain management during subcutaneous venous port (SVP) implantation is essential for patient‐centred cancer care. However, evidence‐based approaches to minimise intraoperative pain remain underexplored. This trial evaluated the clinical effectiveness and safety of the propofol‐alfentanil patient‐controlled sedation (PCS) technique as an adjunct to local anaesthesia (LA) for pain reduction during SVP implantation. Adult cancer patients at two Swedish anaesthesia departments received either LA + PCS or LA alone for SVP implantation. The primary outcome was the maximum intraoperative pain score on an 11‐point numeric rating scale (NRS). Safety outcomes included respiratory, haemodynamic and insertion‐related complications. Secondary outcomes assessed patient satisfaction and procedural measurements. A total of 340 patients (median age 70 [interquartile range, [IQR] 61–76], 51.8% male) were recruited between January 2023 and November 2024. Median intraoperative NRS pain scores were similar between groups (2 [0–3] vs. 2 [0–3], *p* = 0.292), with pain scores ≥ 4 reported in 22.9% (LA + PCS) and 22.2% (LA) (OR 0.96; 95% CI 0.57–1.60; *p* = 0.872). Hypoxia or obstructed airway occurred in 2/166 (1.2%) patients in the LA + PCS group and none in the LA group. Patient satisfaction was high in both groups (10 [10–10], *p* = 0.102). Optimal procedural conditions were reported more frequently with LA + PCS (96.4% vs. 82.0%; OR 5.84; 95% CI 2.36–14.44; *p* < 0.001), without affecting perioperative workflow. Propofol‐alfentanil PCS does not significantly reduce intraoperative pain in patients during SVP implantation. Its routine use for pain reduction cannot be recommended. However, PCS may reasonably be offered to patients who prefer procedural sedation.

**Editorial Comment:**

This randomised clinical trial found that adding patient‐controlled sedation with propofol‐alfentanil to standard local anaesthesia for subcutaneous venous port implantation does not have an impact on pain scores or patient satisfaction. Additional studies focusing on patients experiences and safety are recommended before implementing propofol‐alfentanil patient‐controlled sedation.

**Trial Registration:** EudraCT number: 2021‐003821‐31; ClinicalTrials.gov identifier: NCT 05688384.

## 1 Introduction

Approximately 20 million patients are diagnosed with cancer worldwide each year [1]. The diagnosis presents substantial psychological and physical challenges, rendering patients particularly vulnerable to various aspects of their medical care [2]. A significant proportion of cancer patients are treated with systemic chemotherapy, which is frequently delivered through a totally implanted venous access device (TIVAD), commonly known as a subcutaneous venous port (SVP).

Estimating the total number of SVP implants globally is challenging. In Sweden, with 70.000 cancer diagnoses annually, data from the Swedish Perioperative Registry indicate that approximately 8000 SVPs are implanted each year, making it one of the most frequently performed surgical procedures [3, 4].

The implantation of a SVP is a minor surgical procedure which may elicit pain and anxiety in vulnerable patients. Currently there is no consensus on the optimal procedural sedative or analgesic approach for SVP implantation. Clinical practices vary widely ranging from the use of local anaesthesia (LA) alone, to LA combined with analgosedation or general anaesthesia. In the absence of large‐scale randomised studies on analgesia during SVP implantation, peri‐procedural strategies are frequently guided by institutional traditions rather than evidence‐based recommendations.

Notably, when SVP implantation is performed under LA alone, about 25% of patients experience significant pain and discomfort [5]. The consequences for patients are important and may include refusing a potential re‐procedure under LA, highlighting the necessity of effective analgesic strategies [6].

While clinician‐controlled sedation (CCS) involves a healthcare professional administering analgosedation, patient‐controlled sedation (PCS) represents an autonomous approach, enabling patients to self‐administer and regulate their sedation and analgesia during a procedure according to their own preference [7]. The use of PCS as a technique with various sedative and analgesic agents is well documented and considered a safe alternative [8, 9]. Propofol and alfentanil, due to their short‐acting pharmacological profiles, provide rapid onset and recovery making them particularly suitable for short outpatient procedures.

We conducted the Patient‐controlled Sedation in Port Implantation (PACSPI 2)—a Randomised Clinical Trial to evaluate the effectiveness of PCS using propofol and alfentanil in cancer patients undergoing SVP implantation as adjuncts to LA. We hypothesised that this intervention would reduce the proportion of patients reporting ≥ 4 NRS intra‐procedural pain scores compared to performing the procedure in LA only. Secondary aims were evaluation of safety, patient acceptance, procedural and time measures.

## 2 Methods

### 2.1 Trial Design and Oversight

The PACSPI 2 trial was a pragmatic investigator‐initiated, open‐label, randomised clinical trial conducted at two Anaesthesia departments (Linköping University Hospital and Ryhov County Hospital, Jönköping) in Sweden. The trial was approved by the Swedish Ethical Review Authority (Dnr 2022‐04888‐01/02), the Swedish Medical Products Agency (Dnr: 5.1‐2022‐2057) and was registered on the European Union Clinical Trials Register (EudraCT: 2021‐003821‐31) and clinicaltrials.gov (NCT 05688384) prior to its commencement. This trial followed the Consolidated Standards of Reporting Trials (CONSORT) reporting guideline. Participating sites underwent trial monitoring by an external monitor according to GCP guidelines. A comprehensive data validation of the trial database was performed before commencing analyses. An independent data monitor conducted two safety analyses. The trial protocol is available in the Supporting Information S1.

### 2.2 Participants

Patients aged 18 years or older with a diagnosis of haematological or non‐haematological cancer scheduled for SVP implantation at the centres' Anaesthesia departments were eligible for inclusion. Written informed consent was required of all patients prior to study inclusion. Exclusion criteria were inability to operate the PCS apparatus; inability to communicate in Scandinavian languages; need for general anaesthesia; contraindications to sedation as per anaesthesiologist assessment; non‐fasting status; inability to establish peripheral venous access (PVC); pregnancy or previous enrolment in the trial.

### 2.3 Randomisation

Patients were randomly assigned to two types of analgesic strategies (LA or LA + PCS) in a 1:1 allocation ratio without stratification. The secure, web‐based data capture tool REDCap hosted at Linköping University was used for randomisation, data collection and management [10]. The randomisation sequence was computer‐generated and prepared by an independent statistician using a varying block size of two, four and six. Allocation concealment was maintained but the study intervention was not blinded for either staff or participants.

### 2.4 Interventions

Eligible patients were randomised to either a control group of LA for SVP‐implantation or an intervention group of PCS with propofol and alfentanil as adjuncts to LA for SVP‐implantation.

All patients received an injection of LA in the surgical site, with the type and dosage at the anaesthesiologist's discretion.

Participants randomised to the intervention group were instructed by a nurse anaesthetist on using the PCS pump (Syramed mSP6000; Arcomed AG, Kloten, Switzerland). The syringe contained 36 mL propofol (10 mg/mL) and 4 mL alfentanil (0.5 mg/mL). Each button press delivered 0.5 mL (propofol 4.5 mg/alfentanil 0.025 mg) over 10 s, allowing up to six bolus doses per minute (27 mg propofol and 0.15 mg alfentanil per minute) with no lockout period. Patients could activate the pump immediately after connection to their PVC, prior to preoperative skin antisepsis.

### 2.5 Timing of Assessments

Baseline vital parameters were recorded pre‐procedure, and intra‐procedural monitoring was performed by a nurse anaesthetist. Adverse events (AEs) and serious adverse events (SAEs) were documented for causality and severity (Supporting Information S1). Monitoring included ECG heart rate (HR), non‐invasive blood pressure (BP), oxygen saturation (SpO_2_), and respiratory rate (RR). Bradycardia was defined as HR < 40 bpm/min, tachycardia as HR > 100 bpm/min, hypotension as systolic BP < 90 mmHg or > 30% reduction from baseline, hypoxia as SpO_2_ < 90% or > 5% reduction from baseline, and bradypnea as RR < 8 bpm/min. Due to limited head access with sterile drapes in place, all patients received 2 L/min supplemental oxygen via nasal cannula with capnography monitoring from the start of the procedure.

The Observer's Assessment of Alertness/Sedation score (OAA/S) [11] was used intra‐operatively to determine the sedation level at four pre‐defined procedural steps: (T1) preoperative skin antisepsis; (T2) injection of LA; (T3) catheter tunnelling; and (T4) sterile drape removal. The OAA/S is a 6‐point scale ranging from 5 to 0 that involves eliciting a patient response to increasingly intense stimuli. Response readily to name spoken in normal tone (5); lethargic response to name spoken in normal tone (4); response only after name loudly/repeatedly called (3); response only after mild prodding (2); response only after painful trapezius squeeze (1); no response after painful trapezius squeeze (0).

All SVP implantations were carried out by anaesthesiologists and the operator's experience (< or ≥ 100 procedures) and sex were registered.

The inserting anaesthesiologist assessed operating conditions on a 4‐point scale: (1) procedure can be performed without impact on time or adjustments; (2) the procedure is performed with some impact on time or adjustments; (3) adjustments are required for a procedure of good quality, resulting in longer duration; (4) additional medication is required; the procedure cannot be performed. All SVPs were implanted using the percutaneous Seldinger technique in an operating room setting. Patients were asked to complete a confidential questionnaire before discharge.

### 2.6 Outcomes

#### 2.6.1 Primary Outcome

The primary outcome was maximum pain score during the procedure, assessed using the 11‐point NRS in the recovery unit before discharge (0 = no pain; 10 = worst pain imaginable).

#### 2.6.2 Secondary Outcomes

Several secondary outcomes were evaluated: (1) NRS scores for patient satisfaction with the procedure and pain management (0 = not at all satisfied, 10 = very satisfied); (2) procedural data (vessel, laterality, ultrasound guidance, puncture attempts, time consumption); (3) pharmacological data with delivered doses of propofol, alfentanil and ‘rescue sedation’ (a procedural clinician‐guided response, involving sedation, analgesia, or both), as well as LA type and volume; (4) sedation levels; (5) operating conditions; (6) procedural time data.

Safety outcomes included respiratory, haemodynamic and insertion‐related complications.

### 2.7 Statistical Analysis

Sample size was calculated based on previous data on pain perception during SVP implantation [5, 12]. With an incidence of 25% of patients with a pain NRS score ≥ 4 during SVP implantation in LA, this study aimed to detect a 50% reduction in patients scoring ≥ 4 on NRS in the LA + PCS group. We found this reduction to be clinically meaningful. To detect this difference with 80% power, 5% significance level, two‐sided and with an assumed dropout rate of 10% we aimed to include 340 patients in the study. An independent data monitor oversaw safety for the trial and reviewed data after approximately 100 and 200 patients were enrolled revealing an AE incidence below the pre‐specified 15% threshold. The trial had no predefined stopping criteria for harm, futility or efficacy.

Descriptive statistics were presented with numbers and proportions, median, and interquartile ranges (IQR) as appropriate. IQR was presented with 25th–75th percentiles. Mann Whitney U, Chi Square test of homogeneity and Fisher's Exact test were used to test for differences between groups as appropriate. Analyses on primary and secondary outcomes were performed on intention‐to‐treat (ITT) population. All *p*‐values were two‐tailed, and *p* < 0.05 was considered statistically significant. As the risk of type 1 error is not clearly defined in multiple secondary analyses, analyses for additional outcomes should be treated as exploratory. Statistical analyses were performed using SPSS version 29 (IBM, Armonk, NY, USA).

## 3 Results

### 3.1 Patient Characteristics

**FIGURE 1:**
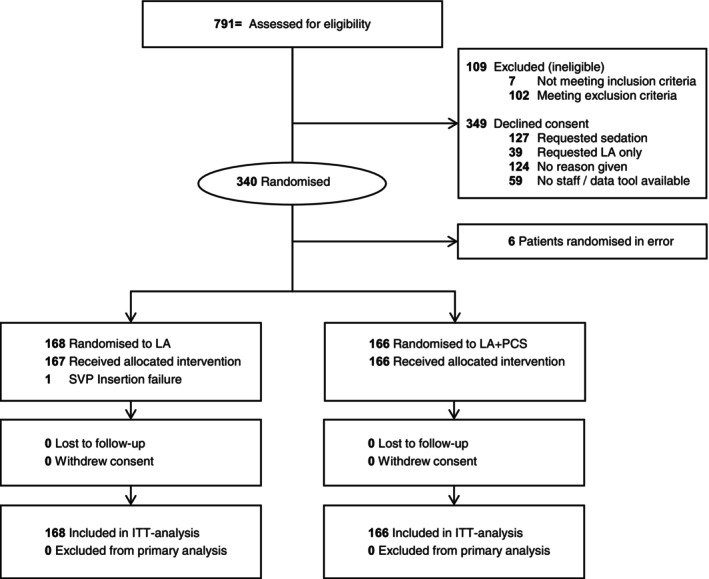
Flow chart CONSORT.

From 19 January 2023 to 8 November 2024 a total of 791 patients were assessed for eligibility. Of these, 109 were excluded, 349 declined participation and 340 were randomised into the study. Six patients were randomised incorrectly meeting exclusion criteria. Of the remaining 334, 168 were assigned to standard care with LA, 166 to the intervention LA + PCS group (Flow Chart, Figure 1). Placement of a SVP was not possible in one patient in the LA group and primary and secondary endpoints were not obtained for that patient. No patient withdrew from the study.

Demographic and clinical characteristics of the patients are shown in Table 1. The median age was 70 (61–76) years, and 173 (51.8%) were male. Demographic and procedure characteristics were similar between the two groups with virtually all catheters being inserted with the help of ultrasound (332, 99.7%) into the right (300, 89.8%) jugular vein (327, 98.2%) (Table 2).

**TABLE 1:** Clinical characteristics of patients.

	LA (*n* = 168)	LA + PCS (*n* = 166)
Age (year), median (min–max), [No]	68 (29–86) [168]	72 (24–89) [166]
Sex, No. (%)	168	166
Female	80 (47.6)	81 (48.8)
Male	88 (52.4)	85 (51.2)
ASA physical status, No. (%)	166	165
1	7 (4.2)	9 (5.5)
2	94 (56.6)	77 (46.7)
3	64 (38.6)	79 (47.9)
4	1 (0.6)	—
BMI, median (IQR), [No]	24.6 (22.7–28.2), [168]	25.1 (22.1–28.3), [165]
HR, median (IQR), [No]	76 (66–84) [168]	74 (65–85) [166]
SaO_2_, median (IQR), [No]	99 (97–100) [168]	99 (97–100) [166]
Syst BP, median (IQR), [No]	139 (128–154) [165]	138 (123–150) [165]
Cancer type No. (%)	167	165
Haematological	14 (8.4)	17 (10.3)
Breast	21 (12.6)	13 (7.9)
Colorectal	35 (21.0)	36 (21.8)
Upper GI‐tract	33 (19.8)	35 (21.2)
Urogenital	32 (19.2)	33 (20.0)
Lung	13 (7.8)	12 (7.3)
ENT	5 (3.0)	2 (1.2)
Other	14 (8.4)	17 (10.3)
Treatment strategy No. (%)	166	165
Adjuvant	82 (49.4)	81 (49.1)
Palliative	84 (50.6)	84 (50.9)
Premedication No. (%)	168	166
No premedication	75 (44.6)	79 (47.6)
Premedication	93 (55.4)	87 (52.4)
Acetaminophen	85 (91.4)	77 (88.5)
NSAID	2 (2.2)	3 (3.4)
Other	6 (6.5)	7 (8.0)
Centre No. (%)	168	166
Jönköping Hospital	86 (51.2)	76 (45.8)
Linköping Hospital	82 (48.8)	90 (54.2)

Abbreviations: ASA, American Society of Anaesthesiology; BMI, body mass index (calculated as weight in kilograms divided by height in metres squared); ENT, ear‐nose‐throat; GI, gastrointestinal; HR, heart rate; IQR, interquartile range; LA, local anaesthetic; max, maximum; min, minimum; *n*, sample size; No, Number; NSAID, nonsteroidal anti‐inflammatory drug; PCS, patient‐controlled sedation; SaO_2_; oxygen saturation; Syst BP, systolic blood pressure; y, years.

**TABLE 2:** Insertion and sedation characteristics.

	LA (*n* = 168)	LA + PCS (*n* = 166)	*p*
Anaesthesiologist sex, No. (%)	168	166	
Male	101 (60.1)	102 (61.4)	
Female	67 (39.9)	64 (38.6)	
Operator experience, No. (%)	168	166	
< 100 SVP	61 (36.3)	67 (40.4)	
≥ 100 SVP	107 (63.7)	99 (59.6)	
Music played in theatre, No. (%)	168	166	
No music	101 (60.1)	99 (59.6)	
Music played	67 (39.9)	67 (40.4)	
Preoperative antibiotics, No. (%)	168	165	
No antibiotics	86 (51.2)	76 (46.1)	
Antibiotics	82 (48.8)	89 (53.9)^a^	
Cloxacillin	74 (90.2)	85 (95.5)	
Vancomycin	3 (3.7)	2 (2.2)	
Other	5 (6.1)	1 (1.1)	
Insertion vein, No. (%)	167	166	
Jugular vein	165 (98.8)	162 (97.6)	
Subclavian vein	2 (1.2)	3 (1.8)	
Axillary vein	—	1 (0.6)	
Laterality, No. (%)	168	166	
Right	144 (85.7)	156 (94.0)	
Left	24 (14.3)	10 (6.0)	
Ultrasound use, No. (%)	168	165	
No ultrasound used	—	1 (0.6)	
Ultrasound used	168 (100)	164 (99.4)	
Catheter Tip position, No. (%)	167	166	
Distal SVC	32 (19.2)	34 (20.5)	
RA	134 (80.2)	131 (78.9)	
Other	1 (0.6)	1 (0.6)	
Catheter type, No. (%)	167	166	
8.5 Fr, small dose	85 (50.9)	88 (53.0)	
8.5 Fr, standard dose	74 (44.3)	69 (41.6)	
Other	8 (4.8)	9 (5.4)	
Local anaesthetic type, No. (%)	168	166	
Mepivacaine 1%	87 (51.8)	83 (50.0)	
Mepivacaine 0.5%	79 (47.0)	83 (50.0)	
Other	2 (1.2)	—	
Adjuvantia, No. (%)	168	166	
Sodiumbicarbonate + Adrenaline	146 (86.9)	141 (84.9)	
Sodiumbicarbonate	11 (6.5)	17 (10.2)	
Adrenaline	11 (6.5)	8 (4.8)	
Local anaesthetic volume (ml), median (IQR), [No.]	30 (20–38) [167]	27 (20–35) [164]	
Puncture attempts, median (IQR), [No.]	1 (1–1) [166]	1 (1–1) [166]	
Operating conditions, No. (%)	167	166	< 0.001
1 Perfect conditions	137 (82.0)	160 (96.4)	
2 Minor impact on conditions	25 (15.0)	6 (3.6)	
3 Major impact on conditions	4 (2.4)	0	
4 Procedure cannot be performed	1 (0.6)	—	
Sedation volume (mL), median (IQR) [No.]	0 (0–0) [165]	6 (3.5–9.5) [166]	< 0.001
Alfentanil delivered mg	0 (0–0)	0.3 (0.17–0.47)	
Propofol delivered mg	0 (0–0)	54 (31.5–85.5)	
Oxygen delivery, No (%)	168	166	
No extra oxygen	29 (17.3)	0	
2 L oxygen/min	137 (81.5)	161 (97.0)	
> 2 L	2 (1.2)	5 (3.0)	
Rescue sedation given No. (%)	168	165	< 0.001
Not given	153 (91.1)	164 (99.4)	
Given	15 (8.9)	1 (0.6)	
Patient requested	10 (66.7)	—	
Operator requested	3 (20)	—	
Other	2 (13.3)	1 (100)	
Rescue sedation type, No. (%)	15	1	
Propofol/Alfentanil	9 (60)	—	
Propofol	4 (26.7)	0	
Alfentanil	2 (13.3)	—	
Other	0	1 (100)	
OAA/S T1, preop skin antisepsis, No (%)	168	166	
5 Awake	167 (99.4)	162 (97.6)	
4 Lethargic to normal tone	1 (0.6)	3 (1.8)	
3 Lethargic to loud tone	—	1 (0.6)	
2 Response mild prodding	0	—	
1 Response painful stimulus	—	—	
0 No response painful stimulus	0	—	
OAA/S T2, local anaesthetic injection, No (%)	168	166	< 0.003
5 Awake	159 (94.6)	141 (84.9)	
4 Lethargic to normal tone	3 (1.8)	19 (11.4)	
3 Lethargic to loud tone	3 (1.8)	5 (3.0)	
2 Response mild prodding	—	0	
1 Response painful stimulus	3 (1.8)	1 (0.6)	
0 No response painful stimulus	—	0	
OAA/S T3, catheter tunnelling No (%)	167	166	< 0.001
5 Awake	155 (92.8)	121 (72.9)	
4 Lethargic to normal tone	6 (3.6)	33 (19.9)	
3 Lethargic to loud tone	3 (1.8)	9 (5.4)	
2 Response mild prodding	—	1 (0.6)	
1 Response painful stimulus	2 (1.2)	—	
0 No response painful stimulus	1 (0.6)	2 (1.2)	
OAA/S T4, drape removal, No (%)	165	163	
5 Awake	163 (98.8)	154 (94.5)	
4 Lethargic to normal tone	—	7 (4.3)	
3 Lethargic to loud tone	1 (0.6)	2 (1.2)	
2 Response mild prodding	—	—	
1 Response painful stimulus	—	—	
0 No response painful stimulus	1 (0.6)	—	
Procedure time min, median (IQR), [No.]	31.5 (25–40) [164]	30.0 (25–36) [165]	0.080
Postop observation time min, median (IQR), [No.]	17.0 (9–32) [167]	16.0 (9–35) [165]	

Abbreviations: Fr, French; L, litre; LA, local anaesthetic; mg, milligram; min, minutes; mL, millilitre; *n*, sample size; No, Number; OAA/S, Observer's Assessment of Alertness/Sedation score; PCS, patient‐controlled sedation; RA, right atrium; SVC, superior vena cava; SVP, subcutaneous venous port.

^a^ Documentation on one antibiotic type was missing.

### 3.2 Primary and Secondary Effectiveness Outcomes

Primary outcome data were available on 167 (99.4%) patients for the LA group, and 166 (100%) for the LA + PCS group. The median pain score on NRS was 2 (0–3) in both groups *p* = 0.292. Pain scores of ≥ 4 on the NRS were reported by 37 patients (22.2%) in the LA group and 38 (22.9%) in the LA + PCS group (OR 0.96; 95% CI 0.57–1.60; *p* = 0.872) (Table 3). Overall satisfaction with the procedure was high in both groups (median 10 (10–10), *p* = 0.753) as was satisfaction with pain management (*p* = 0.102). Patients in the LA + PCS group rated the importance of receiving a sedative during the procedure and being in control of it higher than in the LA group (*p* < 0.001). They also experienced more pain in the arm with the PVC (*p* < 0.001).

**TABLE 3:** Primary and secondary outcomes.

	LA (*n* = 168)	LA + PCS (*n* = 166)	*p*
Maximum pain during procedure No (%)	167	166	0.872
NRS ≤ 3	130 (77.8)	128 (77.1)	
NRS ≥ 4	37 (22.2)	38 (22.9)	
Maximum pain during procedure, median (IQR)	2 (0–3)	2 (0–3)	0.292
Satisfaction overall, median (IQR)	10 (10–10)	10 (10–10)	0.753
Satisfaction with staff, median (IQR)	10 (10–10)	10 (10–10)	0.392
Maximum pain in arm with pvc, median (IQR)	0 (0–1)	1 (0–3)	< 0.001
Importance of getting a sedative, median (IQR)	0 (0–2)	7 (2–10)	< 0.001
Importance to be in control of sedatives, median (IQR)	0 (0–5)	8 (5–10)	< 0.001
Satisfaction with pain management, median (IQR)	10 (9–10)	10 (10–10)	0.102

Abbreviations: IQR, interquartile range; LA, local anaesthetic; *n*, sample size; No, Number; NRS; numeric rating scale; PCS, patient‐controlled sedation; pvc, peripheral venous catheter.

Patients in the LA + PCS group were significantly more sedated at critical procedural steps T2 (OR 0.32; 95% CI 0.14–0.71; *p* < 0.003) and T3 (OR 0.21; 95% CI 0.10 to 0.41; *p* < 0.001) than in the LA group. Rescue sedation was required in 15 patients (8.9%) in the LA group versus one (0.6%) in the LA + PCS group (OR 16.08; 95% CI 2.10–12.183; *p* < 0.001). Procedure time was similar in both groups although the operating anaesthesiologist graded operating conditions as ‘not optimal’ significantly more in the LA group (OR 5.84; 95% CI 2.36–14.44; *p* < 0.001). Time spent in post‐operative care was similar (Table 2).

### 3.3 Safety Outcomes

In the LA + PCS group four patients (2.4%, *p* = 0.06) experienced bradypnea without desaturation; one patient (0.6%) experienced hypoxia treated with increased oxygen delivery; one patient (0.6%) was treated with a chin lift due to an obstructed airway. No respiratory events were noted in the LA group. The incidence of pre‐specified complications and AEs was similar in both groups (Table 4).

**TABLE 4:** Adverse events and complications.

	LA (*n* = 168)	LA + PCS (*n* = 166)	*p*
Accidental arterial puncture, No (%)	168	165	
No	167 (99.4)	163 (98.8)	
Yes	1 (0.6)	2 (1.2)	
Pneumothorax, No (%)	168	166	
No	168 (100)	168 (100)	
Yes	—	—	
Haematoma, No (%)	168	166	
No	167 (99.4)	166 (100)	
Yes	1 (0.6)	—	
Help of Colleague required, No (%)	168	166	0.121
No	162 (96.4)	165 (99.4)	
Yes	6 (3.6)	1 (0.6)	
Procedure aborted, No (%)^a^	168	166	
No	167 (99.4)	166 (100)	
Yes	1 (0.6)	—	
Hypoxia during procedure, No (%)	168	166	
No	168 (100)	165 (99.4)	
Yes	—	1 (0.6)	
Treated by increased oxygen delivery	—	1 (100)	
Bradypnea during procedure, No (%)	168	166	0.060
No	168 (100)	162 (97.6)	
Yes	—	4 (2.4)	
Verbal reminder to breath	—	4 (100)	
Airway check	—	2 (50)	
Obstructed	—	—	
Chin lift	—	1 (25)	
Obstructed airway observed	168	166	
No	168 (100)	165	
Yes	—	1 (0.6)	
Verbal instruction PCS	—	1	
Chin lift	—	1	
Maskventilation necessary, No (%)	168	166	
No	168 (100)	166 (100)	
Yes	—	—	
General anaesthesia needed, No (%)	168	166	
No	168 (100)	166 (100)	
Yes	—	—	
Tachycardia, No (%)	168	166	
No	161 (95.8)	158 (95.2)	
Yes	7 (4.2)	8 (4.8)	
Bradycardia, No (%)	168	166	
No	168 (100)	166 (100)	
Yes	—	—	
Hypotension, No (%)	168	166	
No	168 (100)	166 (166)	
Yes	—	—	
Nausea, No (%)	168	165	
No	166 (98.8)	165 (100)	
Yes	2 (1.2)	—	

Abbreviations: LA, local anaesthetic; *n*, sample size; No, Number; PCS, patient‐controlled sedation.

^a^ One procedure was aborted due to anatomical reasons.

## 4 Discussion

In this pragmatic trial of 334 participants with cancer undergoing SVP implantation, the adjunct of propofol‐alfentanil PCS did not reduce pain perception compared to LA alone. Complications and adverse events were similar in both groups. Participants in both groups were highly satisfied with their care and pain management. Almost 10% of the patients received rescue sedation in the standard care group. Although operating conditions were graded better in the intervention group, suggesting potential workflow benefits, intra‐ and post‐operative time measures did not differ.

Since its first description [7], the concept of PCS, involving self‐administered analgosedation has become more widespread and is an accepted patient‐tailored approach for short procedures in various clinical contexts [8, 13]. Sedative and analgesic regimens vary and data on respiratory safety are conflicting. PCS with a combination of propofol and alfentanil was shown to facilitate completion of gynaecological operative procedures compared with propofol alone; however, respiratory status was compromised. In contrast, it was found to have no adverse respiratory effects in the setting of endoscopic retrograde cholangiopancreatography [13, 14]. The regimen of propofol and alfentanil in this trial was suggested to be safe in our previous feasibility trial [15]. Results in the current trial align with these findings showing no statistically significant respiratory complications in the intervention group. However, one patient with hypoxia and one patient with an obstructed airway mandate the need for adequate monitoring, staffing and setting during the procedure, as recently emphasised in a competency framework [16].

One single centre RCT evaluated the efficacy of various remifentanil doses, administered as target controlled infusion (TCI), in combination with propofol for both tunnelled catheter insertion and removal [13]. While higher doses provided comparable analgesia, they were associated with increased respiratory complications. However, since catheter insertion and removal probably have different analgesic requirements, these findings are difficult to apply to SVP insertion procedures only.

Another single centre RCT comparing midazolam fentanyl PCS to CCS for insertion of long‐term CVC's reported low pain scores [17] whereas our findings showed no analgesic benefit of LA + PCS over LA alone. Differences in results may be due to a small sample size (*n* = 40), use of longer‐acting fentanyl, and the limited number of SVP insertions in that study. Postoperative observation time in our trial was brief and similar in both groups compared to the two‐hour stay in the referenced study, highlighting the advantage of short‐acting medications if analgosedation is required [17].

Importantly, previous studies examined different systemic analgesic strategies as adjuncts to LA but did not include SVP implantation under LA alone as a comparator. One interview study reported low pain scores for SVP implantation with LA alone [18], consistent with our findings of a median NRS score of 2. This suggests that routine administration of additional systemic analgesia may not be necessary and could lead to overtreatment and increased costs. However, reliance on median pain scores may underestimate the fact that a considerable proportion of patients still experience significant pain.

Self‐administered doses of analgosedation varied widely (0–23 mL), highlighting substantial interindividual differences in sedation and analgesia needs. The administration of rescue sedation in the LA group confirms this variability and is consistent with previous findings [17]. Providing analgosedation to patients who do not activate the PCS pump results in unnecessary medication costs. Considerable variability in anxiolytic preferences for SVP insertion has been reported previously, supporting the use of decision aids to align perioperative care with patient goals [19]. While our trial focused on pain, other factors—such as anxiolysis, emotional comfort, and avoidance of unpleasant recall—may be equally important but were not assessed. Over a hundred patients declined participation as they preferred analgosedation as per clinical routine during the procedure. These choices may reflect anxiety, concerns about pain, or prior unpleasant experiences. Predicting individual sedation requirements in advance is important and challenging, emphasising the role of patient self‐assessment and shared decision‐making in tailoring analgosedative strategies to individual needs. For those desiring additional analgosedation, PCS may be a suitable option.

Interestingly, patients in both groups reported high satisfaction with their pain management and peri‐procedural care. Measuring patient satisfaction remains challenging in the absence of validated patient‐related experience measures for this kind of procedure. Interpretation is further complicated by a pronounced ceiling effect and the inherently broad, subjective nature of satisfaction, which is influenced by numerous non‐clinical factors. These findings underscore that pain assessment alone is too one‐dimensional and only partially contributes to the overall patient experience, a phenomenon well documented in postoperative pain research [20, 21]. Our findings are consistent with previous studies evaluating various analgesic strategies for SVP implantation [17, 18, 22]. Qualitative research on PCS emphasises the critical role of patient‐healthcare professional interactions in shaping the overall experience [23]. Factors such as patient‐centred communication and trust, may contribute to lower pain perception during medical care and significantly influence overall satisfaction scores [24, 25]. This underscores the importance of the patient‐staff relationship before and during the SVP procedure.

Our trial has several strengths. Its large sample size was well suited to address the primary endpoint, while broad inclusion criteria and a multicentre design enhance the generalisability of the findings within similar health care systems and settings. Additionally, the inclusion of safety endpoints ensures a high standard of patient safety data for this intervention, meeting criteria for a pragmatic RCT [21].

However, important limitations must be acknowledged. First, the choice of pain perception as the primary outcome captures only one dimension of the patient experience, leaving other relevant dimensions such as anxiety, emotional comfort, and unpleasant recall, unassessed. Pain evaluation in the recovery room may suffer from recall bias, especially in patients in the LA + PCS group, making interpretation challenging. Second, although adverse events did not significantly differ in the LA + PCS group the trial was not powered for rare but potentially serious safety events. Third, among those who declined participation, one‐third requested sedation, leading to a potential selection bias as a patient group that might benefit most from PCS was excluded despite the randomised design. Finally, the lack of concealment in group allocation and intervention increases the risk of bias, particularly for subjective outcomes such as pain perception and operating condition ratings. Participants' awareness of treatment assignment may have influenced perceived effectiveness, potentially introducing placebo or nocebo effects that could distort results. In addition the use of a non‐validated questionnaire for assessing patient experience limits the reliability of subjective outcome measurements.

## 5 Conclusion

Propofol‐alfentanil PCS does not reduce intra‐procedural pain in patients undergoing SVP implantation. Its routine use for pain reduction cannot be recommended. However, PCS may reasonably be offered to patients who prefer additional sedation for the procedure.

Future research should focus on sedation preference predictors, for example preoperative anxiety scales, structured questionnaires or qualitative approaches in order to better understand patient perspectives and identify those who would truly benefit from PCS.

## Author Contributions

Conception and design: Stefanie Seifert, Knut Taxbro and Fredrik Hammarskjöld. Statistical analysis: Stefanie Seifert and Knut Taxbro. All authors contributed to acquisition of data, analysis and interpretation of data, drafting the article or revising it critically for important intellectual content, final approval of the version to be published and accountability for all aspects of the work.

## Disclosure

Futurum approved the protocol. Futurum did not have a role in the design, study conduct, data collection, management, analysis, and interpretation of the data; preparation, review, or approval of the manuscript, and decision to submit the manuscript for publication. Data collection, management, data analysis, and preparation of the manuscript were conducted and managed by the corresponding author at the Department of Anaesthesia and Intensive Care Medicine, Ryhov County Hospital, Jönköping, Sweden. Supervision: Trial monitoring was carried out by Forum Östergötland according to GCP guidelines. FORUM facilitated data oversight, compliance tracking, and quality assurance throughout the study. The authors retain full responsibility for the study design, data analysis, and interpretation of results.

## Ethics Statement

The trial was approved by the Swedish Ethical Review Authority (Dnr 2022‐04888‐01/02) 2022‐02‐08, the Swedish Medical Products Agency (Dnr: 5.1‐2022‐2057) and was registered on the European Union Clinical Trials Register (EudraCT: 2021‐003821‐31) and clinicaltrials.gov (NCT 05688384) prior to its commencement. The trial was conducted in compliance with the standards of Good Clinical Practice (GCP) defined by the International Council for Harmonisation of Technical Requirements for Pharmaceuticals for Human Use (ICH), ethical principles outlined in the Declaration of Helsinki, and all relevant national regulations.

## Consent

Written information was provided and informed consent was required of all patients prior to study inclusion.

## Conflicts of Interest

The authors declare no conflicts of interest.

## Supporting information


**Data S1:** Supporting Information.

## Data Availability

The data that support the findings of this study are available from the corresponding author upon reasonable request.
