# Supplementary material for: Patient‐Controlled Sedation in Port Implantation (PACSPI 2)—A Randomised Clinical Trial
Source: Acta Anaesthesiol Scand. 2025 Nov 10;70(1):e70148. doi: 10.1111/aas.70148 (PMC12602766; doi:10.1111/aas.70148)
Supplement: Supplementary file 1 — Data S1: Supporting Information. [file AAS-70-0-s001.pdf]

Statistical Analysis Plan PACSPI 2

TRIAL FULL TITLE	Patient-controlled Sedation in Port Implantation (PACSPI_2) - a randomized controlled trial
EPM Nr<br>EPM amendment Nr	EPM: 2022-00088-01<br>EPM: 2022-00088-02
EUDRACT NUMBER	2021-003821-31
SAP VERSION	1.0
STUDY CODE	Futurum-963747
SAP VERSION DATE	2022-03-17
TRIAL STATISTICIAN	Bo Rolander
TRIAL SPONSOR	Knut Taxbro
TRIAL PRINCIPAL INVESTIGATOR	Stefanie Seifert
SAP AUTHOR	Stefanie Seifert

1 SAP Signatures

I give my approval for the attached SAP entitled **Patient-Controlled Sedation in Port Implantation (PACSPI-2**) **-a randomized controlled trial** dated Monday, 3rd october 2022. I assure that this trial will be conducted in compliance with all stipulations of this statistical analysis plan, the conditions in the Swedish Ethical Review Authority and Swedish medical products agency approval, standards of Good Clinical Practice (defined by the International Conference on Harmonisation), ethical principles that have their origin in the Declaration of Helsinki and all applicable national and local regulations.

Trial Sponsor
Name: Knut Taxbro

Signature: ________________________

Date: ________________________

Principal Investigator
Name: Stefanie Seifert

Signature: ________________________

Date: ________________________

Trial Steering Group

Trial Sponsor
Knut Taxbro
OP/IVA kliniken
Ryhov County Hospital
55185 Jönköping
Email: knut.taxbro@rjl.se>

Prinicipal Investigator
Stefanie Seifert
OP/IVA kliniken
Ryhov County Hospital
55185 Jönköping
Email: stefanie.seifert@rjl.se>

Co-Investigator
Josip Azman
An/OP/IVA kliniken
Linköping University Hospital
Email: Josip.Azman@regionostergotland.se>

Co-Investigator
Andreas Nilsson
An/OP/IVA kliniken
Linköping University Hospital
Email: Andreas.Nilsson@regionostergotland.se>

Co-Investigator
Michelle Chew
An/OP/IVA kliniken
Linköping University Hospital
Email: Michelle.Chew@regionostergotland.se>

Co-Investigator
Fredrik Hammarskjöld
OP/IVA kliniken
Ryhov County Hospital
55185 Jönköping
Email: fredrik.hammarskjold@rjl.se>

Statistician
Bo Rolander
Futurum
Ryhov County Hospital
55185 Jönköping
Email: bo.rolander@rjl.se>

Monitor:
FORUM Östergötland
Region Östergötland
Universitetssjukhuset I Linköping
58185 Linköping
forumo@regionsostergotland.se

Study Summary:

Title: Patient-Controlled Sedation in Port Implantation (PACSPI 2) – a randomized controlled trial.

Design: A randomized controlled trial at two centres with a total of 340 patients.

Aims: To determine if patient-controlled sedation (PCS) with propofol and alfentanil reduces patient-reported pain perception during implantation of subcutaneous venous port (SVP).

Outcome Measures: The primary outcome is pain perception. Secondary outcomes include patient satisfaction and efficacy measures such as periprocedural time consumption, sedation levels, insertion conditions as well as safety measures, adverse events and quality of recovery.

Population / Eligibility: Patients ≥18 years with cancer in need of SVP.

Exclusion criteria:

- Inability to operate the PCS apparatus.
- Inability to communicate in Scandinavian languages.
- Patients who require general anaesthesia or patients eligible for LA only on anesthesiologist´s assessment (i.e. severe sleep apnea).
- Propofol or alfentanil allergy.
- Non-fasting according to guidelines of the Swedish Society for Anaesthesia and Intensive Care (SFAI).
- Failure to achieve peripheral vascular access.
- Pregnancy
- Previous participation in study

Trial medication: Propofol 10mg/ml**,** Alfentanil 0,5mg/ml. A syringe loaded with 36ml Propofol and 4ml Alfentanil in a patient controlled sedation (PCS) pump with 0.5ml aliquots per pressed handheld button.

Duration of Trial Participation: Participation in the trial will continue until the patient is discharged from the postoperative unit having completed the patient questionnaire. For measurement of quality of recovery a telephone interview will be performed one day postoperatively.

Study period: Q 4 2022-Q3 2024.

2 Table of Contents

1 SAP Signatures ... 1
2 Table of Contents ... 4
3 Abbreviations and Definitions ... 5
4 Introduction ... 7
4.1 Existing Research and pilot studies ... 8
4.2 Purpose of the analyses ... 8
4.3 Study Objectives ... 8
5 Study Methods ... 10
5.1 General Study Design and Plan ... 10
5.2 Method ... 11
5.3 Inclusion-Exclusion Criteria and General Study Population ... 12
5.4 Enrolment, Flow sheet, Randomization and Blinding ... 12
5.4.1 Enrolment ... 12
5.4.2 Randomization ... 13
5.5 Withdrawal Criteria ... 13
5.5.1 Blinding ... 13
5.6 Study Variables ... 14
6 General Considerations ... 19
6.1 Data requisition ... 19
6.2 Timing of Analyses ... 19
6.3 Analysis Populations ... 19
6.3.1 Full Analysis Population ... 19
6.3.2 Per Protocol Population ... 19
6.3.3 Intention to Treat Analysis ... 19
6.4 Subgroups ... 19
6.5 Missing Data ... 19
6.6 Interim Analyses and Data Monitoring ... 20
6.6.1 Interim Analyses and Data Quality ... 20

6.6.2 Stopping Rules 20
6.6.3 Bias 20
6.7 Multi-centre Studies 20
6.8 Multiple Testing 20
7 Summary of Study Data 21
7.1 Data collection 21
7.2 Derived variables 21
7.3 Electronic case report form (e-CRF) 21
7.4 Follow-Up and End of Trial 22
8 Study Management 22
8.1 Trial Steering Group (TSG) 22
8.2 Data and Safety Monitoring Board (DSMB) 22
8.3 Annual Development safety Update Report, (DSUR) 22
8.4 Ethical considerations 22
9 Swedish Medical Products Agency and European Medical Agency 23
10 Adverse Events 23
10.1 Definition AE, SAE and SUSAR 23
10.2 Causality and severity of AE and SAE 24
11 Statistics and Data Analysis 25
11.1 Subject Disposition 25
11.2 Protocol Deviations 25
11.3 Demographic and Baseline Variables 26
11.4 Prior and Concurrent Medications 26
11.5 Treatment Compliance 26
11.6 Pregnancies 26
12 Patient satisfaction data 26
13 Patient perioperative experience and recovery 26
14 Confidentiality 26
15 Figures 27
16 Reporting Conventions 28

17 Funding 28
18 Listing of Tables, Listings and Figures 28
19 References 30
20 Supplements 31

3 Abbreviations and Definitions

AE	Adverse Event
ASA	American Society of Anesthesiologists
CCS	Clinician-controlled sedation
CONSORT	Consolidated Standards of Reporting Trials
CRF	Case Report Form
CWF	Consent Withdrawal Form
DSMB	Data and Safety Monitoring Board
EPM	Etikprövningsmyndigheten, Swedish Ethical Review Authority.
$GABA_A$	γ-Aminobutyric Acid A
GCP	Good Clinical Practice
GDPR	General Data Protection Regulation
ICH	International Council for Harmonisation
ITT	Intention-to-treat
LA	Local Anaesthesia
LMV	Läkemedelsverket, Swedish medical products agency
LVLS	Last visit last subject
NRS	Numeric Rating Scale
OAA/S	Observer Assessment of Alertness / Sedation Scale
PCS	Patient-controlled sedation
QoR-15 swe	Quality of Recovery-15 swedish
RCS	Radiologist-controlled sedation
RCT	Randomized Controlled Trial
SAE	Serious Adverse Event
SFAI	Swedish Society for Anaesthesia and Intensive Care
SPOR	Swedish Perioperative Register
SVP	Subcutaneous Venous Port
SUSAR	Suspected Unexpected Serious Adverse Reaction
TIVAD	Totally Implantable Venous Access Device
TSG	Trial Steering Group

4 Introduction

More than 60.000 patients are diagnosed with cancer every year in Sweden. Many of these patients are eligible for chemotherapy through a totally implanted venous access device (TIVAD) commonly referred to as a subcutaneous venous port (SVP). According to the Swedish Perioperative Register [1] the implantation of SVPs is one of the most common surgical procedures in Sweden. However there is no current guidance as to which procedural analgesic strategy is superior during SVP-implantation.

The implantation procedure can be performed in local anaesthesia (LA) only, LA in combination with analogosedation or under general anaesthesia. Practice is likely to be based on local institutional traditions rather than evidence-based guidance.

During SVP implantation using LA alone one fourth of patients experience severe pain and discomfort often leading to administration of sedatives and analgesics for pain relief [2].

Clinician-controlled sedation (CCS) involves administration of procedural analgosedation by a trained clinician. However, it carries the risk of oversedation and generates higher costs compared to alternative sedation methods [3, 4]. Patient-controlled sedation (PCS) is an alternative sedation method to CCS, enabling patients to self-administer and self-regulate their sedation and analgesia during the procedure. The procedural use of PCS with different sedatives and analgesics is well described and regarded as a safe alternative with a lower incidence of analgesic or sedative rescue interventions compared to CCS in a number of clinical settings [5]. Propofol and alfentanil, with their short-acting properties, ensure rapid induction and recovery, making them suitable for outpatient procedures.

The optimal procedural sedative and analgesic strategy regarding the care for patients in need of SVP-implantation is unclear and to the best of our knowledge no randomized controlled trial (RCT) has assessed PCS with propofol and alfentanil as adjunct to LA during SVP-implantation.

A patient’s ability to resume normal activities after surgery and anaesthesia is an important indicator of a successful perioperative experience. Quality of recovery-15 (QoR-15) is a validated psychometric patient-centered outcome measure after day case surgery. QoR-15 has not been used in patients undergoing SVP implantation. This study will assess patients´ perioperative experience by using QoR-15.

In this multicenter RCT we aim to examine the effect of PCS with propofol and alfentanil on patients´ self-reported pain perception scores, overall satisfaction scores, sedation scores and incidence of adverse events.

4.1 Existing Research and pilot studies

Review of the literature
Searching for publications in the literature is challenging due to widespread terminology of TIVAD´s. No less than ten different synonyms were identified (central venous port catheter; subcutaneous venous port; totally implantable access device; totally implantable venous access system; port-a-cath, totally implantable access port; long term central venous catheters, central venous access device, totally implantable subcutaneous port, subcutaneously implanted port-chamber catheter, implantable chest wall port). All synonyms are aiming at the same device- a subcutaneously buried device connected to a catheter with its tip in a large bore vein.
We conducted a review of the literature.

Pubmed was searched up to 10th March 2022 for randomized controlled trials.

We searched pubmed with the above mentioned synonyms AND patient-controlled sedation. 1 RCT with 40 patients was identified comparing effects between PCS and radiologist-controlled sedation (RCS) during tunnelled central line insertion [6]. The applied medication being midazolam and fentanyl in both PCS and RCS.

Search for the above mentioned synonyms AND sedation AND adults resulted in 8 studies of which 3 are designed as RCT´s [6-8].

4.2 Purpose of the analyses

These analyses will assess the efficacy of patient-controlled sedation on reduction of patient-reported pain and distress during subcutaneous venous port insertion. It will measure patients` perioperative experience.

4.3 Study Objectives

The primary objective is to determine the efficacy of patient-controlled sedation (PCS) as adjunct to local anaesthesia (LA) on patients´ self-reported pain perception during SVP-implantation. Patients in the treatment arm will undergo SVP insertion in LA combined with PCS and in the control arm SVP insertion will be conducted in LA.

The second objective is analyses of patient distress and satisfaction as well as secondary efficacy outcomes and determination of the relative safety of the treatment arm on defined safety outcomes and all adverse events and costs.

Primary endpoint: The primary endpoint is assessment of patient-reported pain perception. Endpoints are assessed by the use of the numeric rating scale (NRS):

- maximal intraprocedural pain level on NRS

Secondary endpoints:

- satisfaction with pain treatment during implantation
- maximal distress level
- importance of receiving sedatives during implantation
- overall satisfaction
- overall satisfaction with the staff
- maximal pain from arm with PCS infusion
- importance of being in control of sedation administration
- grading of implantation conditions by the implanting physician
- perioperative and procedural time consumption
- delivered doses of alfentanil and propofol
- rescue-therapy by nurse
- differences in cost
- vital parameters: oxygen saturation, heart rate, blood pressure
- sedation levels according to Observers Assessment of Alertness/Sedation scale (OAA/S)
- procedural data,
- Quality of Recovery-15 (QoR-15)

Safety endpoints:

- arterial puncture
- pneumothorax
- hypotension
- arrhythmia
- hypoxia
- airway intervention
- respiratory rate

5 Study Methods

5.1 General Study Design and Plan

The study is an open multicentre randomized controlled trial with a study and a control arm in a 1:1 ratio. Patients will be randomized to either a control arm of LA for SVP-implantation or a study arm of PCS with propofol and alfentanil as adjunct to LA for SVP-implantation. The aim is to randomize 340 patients with an estimated patient recruitment over 18 months with a following 6 months of data cleaning and analysis. The trial will be performed at two centres; County Hospital Ryhov, Jönköping, Sweden and University Hospital Linköping, Sweden. SVP implantation procedure and perioperative time period is estimated to 2-4 hours. The primary endpoint is assessment of patients´ self-reported pain perception. Secondary outcomes include patient satisfaction, implantation conditions, sedation level, sedative and analgesic medication consumption, procedural time consumption as well as safety aspects, adverse events and estimation of perioperative experience. Participation in the trial ends after telephone inquiry one day postoperatively.

Control arm:

SVP-implantation in LA:

Subcutanous venous ports were introduced in 1981. A SVP is a small device around 3cm in diameter with an injectable membrane buried just under the skin. It is connected to a thin tube with its tip in a large bore vein. Access to the SVP is achieved by puncturing the skin with a needle.
Local anaesthetic causes the absence of pain sensation in the location where applied by decreasing the rate of depolarization and repolarization of excitable membranes such as nociceptors and nerves. The most commonly used solutions are from the amide group differing in pharmacokinetics. In order to reduce a burning sensation on infiltration substances can be combined with sodium bicarbonate. There will be no restrictions in the choice of LA-solution being left up to local practice at participating sites. With the beginning of the procedure LA is subcutaneously applied to patients in both groups. LA used at participating centres are Mepivacaine 10mg/ml, 20-40ml subcutaneously or Lidnocaine 10mg/ml, 20-40 ml subcutaneously.

Study arm:

SVP-implantation in LA and PCS:

In addition to LA, patients in the study group are able to self-administer a combination of propofol and alfentanil using a patient-controlled sedation pump. The pump enables the patient via a hand-held button to trigger the release of a single bolus of 0.5ml containing 4.5mg propofol and 25µg alfentanil under an 8 second period. This results in a maximal possible amount of 7 bolus doses per minute.

Propofol is a short-acting anaesthetic agent commonly used for general anaesthesia and procedural sedation. Several mechanisms of action have been proposed both through potentiation of $GABA_A$ receptor activity and in higher doses behaving as $GABA_A$ receptor agonist. When used for intravenous sedation a single dose wears off within minutes demanding for continuous or intermittent application.
Alfentanil is a short-acting synthetic opioid analgesic agent with rapid onset of effects at μ-opioid receptors. These properties make it suitable to provide analgesia for brief procedures and to infuse for longer procedures and yet provide relatively rapid recovery.

Rescue sedation will be available to patients in both groups on the patient´s or operator´s demand. Rescue sedation consists of propofol and alfentanil administered by the clinician and is documented in the e-CRF.

Setting:
This trial will be carried out in the following anaesthesia departments:
OP/IVA-klinken, County Hospital Ryhov, Sweden
AnOpIVA-kliniken, University Hospital Linköping, Sweden

5.2 Method

Participants will be instructed on use of the PCS pump (Syramed μSP6000, Arcomed AG, Switzerland) by a nurse anesthetist. The syringe is loaded with 36 ml propofol (10 mg/ml) and 4 ml alfentanil (0.5 mg/ml). Each time the patient presses the handheld button, an aliquot of 0.5 ml is injected (4.5 mg propofol/0.025 mg alfentanil). The injection time is set to 8 s, restricting self-administration to a maximum of 7 bolus doses per minute corresponding to 31.5 mg propofol and 0.175 mg alfentanil per minute. No lockout period is applied. Local anaesthesia is injected in the operating site. Vital parameters prior to the procedure are recorded. Patients are monitored using electrocardiography for heartrate (HR), non-invasive blood pressure (BP), oxygen saturation (SpO_2), and respiratory rate (RR) at 5-min intervals during the procedure. Bradycardia is defined as HR <40 beats/min, tachycardia as HR >100 beats/min, hypotension as systolic BP < 90 mmHg or a decrease of >30% from baseline, hypoxia as SpO_2 <90% or a decrease of >5% from baseline, and bradypnea as RR of <8 breaths per minute. Supplemental oxygen via a capnograph-fitted nasal cannula is administered to all patients at 2 L/min during the procedure. The Observer`s Assessment of Alertness/Sedation score (OAA/S) [9] is used to determine the sedation level during the four procedural steps: 1) sterile swabbing, 2) injection of LA, 3) catheter tunneling, and 4) sterile drape removal. The operating anesthesiologist assesses the operating conditions on a 4 point scale with 1 enabling the operator to perform the procedure without time delay and 4 having to abort the procedure. Puncture attempt is defined as continuous needle advancement to establish vein puncture. An unvalidated patient perception assessment tool with seven dimensions applying the NRS is used to evaluate patient perception. QoR-15 will be measured att 3 timepoints. Preoperatively, postoperatively before patient discharge and one day postoperatively.

5.3 Inclusion-Exclusion Criteria and General Study Population

Inclusion criteria:

- Adult patients (≥18 years) with cancer scheduled for SVP-implantation at participating anaesthesia departments.

Exclusion criteria:

- Inability to operate the PCS apparatus,
- Inability to communicate in Scandinavian languages.
- Patients who require general anaesthesia or patients eligible for LA only on anesthesiologist´s assessment (i.e. severe sleep apnea).
- Propofol or alfentanil allergy. *
- Non-fasting according to guidelines of the Swedish Society for Anaesthesia and Intensive Care (SFAI).
- Failure to achieve peripheral vascular access.
- Pregnancy **
- Previous participation in study

Anaesthesiology assessment will be conducted at the following stages: SVP request, trial inclusion and preoperatively. Assessment includes co-medication assessment.

*According to Summary of Product Characteristics (SmPC) the following is regarded as contraindication:
Propofol: allergy or hypersensitivity against propofol or any of the excipients of the emulsion, Patients who are allergic to peanut or soya.
Alfentanil: hypersensitivity to the active substance, to other opioids, or to any excipients listed under section: List of excipients in SmPC.

**Women in fertile age will be able to participate in the trial after pregnancy testing at the oncology unit before inclusion into the trial (according to Recommendations related to contraception and pregnancy testing in clinical trials).

5.4 Enrolment, Flow sheet, Randomization and Blinding

5.4.1 Enrolment

Eligibility for enrolment will be judged by the SVP-scheduling nurse according to inclusion and exclusion criteria. Screening log will be established.
Patients will be given an information sheet about the possibility to participate at the oncology / hematology department prior to SVP booking. On arrival to the preoperative unit a study team member will give full explanation and a physician will be available to answer questions. Signed informed consent is obtained by a physician on the day of the procedure. Inclusion

and randomization and treatment are only applied after the patient not meeting any exclusion criteria. All participants are free to withdraw at any time from the protocol treatment without giving reasons and without prejudicing further treatment (section 5.5).

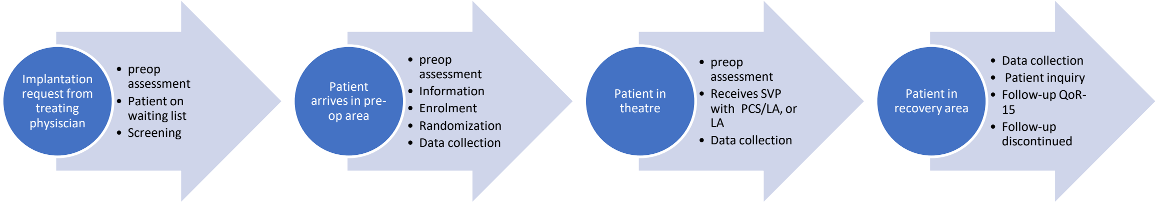

5.4.2 Randomization

Prior to SVP-implantation and when the patient´s eligibility has been confirmed and consent forms have been completed the patient will be randomized by password-coded randomization software accessible to the co-investigators at each participating site (REDCap). All patients must be randomized onto the study prior to SVP-implantation.

Each patient randomized will be allocated a unique sequential patient code number for the randomization arm together with an allocated study arm.

The randomization scheme will be equal allocation (1:1) using an electronic randomization tool. Randomization will be performed consecutively. Patients will not be able to be randomized to the study until all appropriate regulatory requirements have been completed. If a patient withdraws from the trial the unique patient code will not be used again and the patient will not be included into the trial again.

5.5 Withdrawal Criteria

Patients will be informed that they have the right to withdraw from the study at any time for any reason, without prejudice to their medical care. Full details of the reasons for withdrawal will be recorded on the Case Withdrawal Form (CWF) which must be completed if a patient wishes withdrawal from the study and send to the principal investigator. By withdrawal from the study previously collected data remains part of the study database. No subsequent data will be used after withdrawal.

5.5.1 Blinding

Due to the nature and effects of sedative medication, study participation is not blinded for neither staff nor participants.

5.6 Study Variables

Variable number	Variable source	Variabel beskrivning	Variable name	Variable code	Variable characteristics	presentation	Analysis	Registration time /implantation
**1.**	CRF	QoR-15 pre	QoR-15 pre	0-10	ordinal	Manus 2	n, Median, interkvartil	CRF
**2.**	CRF	Trial subject nr	PtID	1-340	continuous			pre
**3.**	CRF	gender	gender	1=female<br><br>2=male	categorical/nominal	Table 1	% x/n	pre
**4.**	CRF	age	age	Number 18-105	continuous	Table 1	n, Median, min max, IQR	pre
**5.**	CRF	ASA	ASA	Number 1-5	ordinal	Table 1	P% x/n	pre
**6.**	CRF	weight	weight	30-250	continuous	Table 1	n, Median, min max, IQR	pre
**7.**	CRF	length	length	1,3-2,1	continuous	Table 1	n, Median, min max, IQR	pre
**8.**	CRF	Treatment strategy	strat	1= palliative<br><br>0=adjuvant	categorical/nominal	Table 1	P% x/n	pre
**9.**	CRF	patient arrival duva	Time duva	Hh:mm	continuous	Table 1	Derive preop tid<br><br>Median, min max, IQR	pre
**10.**	CRF	patient arrival or	Time op	Hh:mm	continuous	Table 1	Derive preop tid<br><br>Median, min max, IQR	pre
**11.**	CRF	heartrate	HR	30-150	continuous	Table 1	n, Median, min max, IQR	pre

**12.**	CRF	Saturation	SaO2	50-100	continuous	Table 1	n, Median, min max, IQR	pre
**13.**	CRF	BP systolisk	BT	60-230	continuous	Table 1	n, Median, min max, IQR	pre
**14.**	CRF	Laterality port	side	1= right<br>0=left	categorical/nominal	Table 2	P% x/n	pre
**15.**	CRF	Cath type	Cat type	0=BBraun<br>1=other	categorical/nominal	Table 2	P% x/n	pre
**16.**	CRF	Local anaesthetic volume	LA_vol	0-100 i ml	continuous	Table 2	n, Median, min max, IQR	intra
**17.**	CRF	Local anaesthetic type	LA type	0=carbocaine<br>1=lidnocaine<br>2=other	categorical/nominal	Table 2	P% x/n	intra
**18.**	CRF	LA adjuvans	LA	0=nej<br>1=natriumbikarbonat<br>2=adrenaline<br>3=nabi and adrenaline	categorical/nominal	Table 2	P% x/n	intra
**19.**	CRF	Vein	vein	0=v jugularis int<br>1=v subclavia<br>2=v femoralis	categorical/nominal	Table 2	P% x/n	intra
**20.**	CRF	Ultrasound guidande	US	1=yes<br>0=no	categorical/nominal	Table 2	P% x/n	intra
**21.**	CRF	Puncture number	punctnumber	1-20	continuous	Table 2	n, Median, min max, IQR	intra
**22.**	CRF	Arterial puncture	apunct	0=no<br>1=yes	categorical/nominal	Table 2	P% x/n	intra
**23.**	CRF	Pneumothorax	pnthx	0=no<br>1=yes	categorical/nominal	Table 2	P% x/n	intra
**24.**	CRF	Hematoma operating site	haematom	0=no<br>1=yes	categorical/nominal	Table 2	P% x/n	intra
**25.**	CRF	Operator	operator	0 = ≤ 19 /year<br>1 = 20-49 /year	categorical/nominal	Table 2	P% x/n	intra

				2 = ≥ 50 / year				
**26.**	CRF	Help of colleague	collegue	0=no<br>1=yes	categorical/nominal	Table 2	P% x/n	intra
**27.**	CRF	Operating conditions	Op_cond	1-4	ordinal	Table 2	P% x/n	intra
**28.**	CRF	Aborted procedure	aborted	0=no<br>1=yes	categorical/nominal	Table 2	P% x/n	intra
**29.**	CRF	Position tip	Tip_pos	1=right atrium<br>2=distal v subclavia<br>3=other	categorical/nominal	Table 2	P% x/n	intra
**30.**	CRF	Op time	Op_time	minutes	continuous	Table 2	n, Median, min max, IQR	intra
**31.**	CRF	Sedation<br>score at<br>T1-4<br>(OAAS)	sedT1<br>sedT2<br>sedT3<br>sedT4	OAAS scale (0-5)	ordinal	Table 3	P% x/n	intra
**32.**	CRF	Sedation volym	DelVOL	Volume PCS ml	continuous	Table 3	n, Median, min max, IQR	intra
**33.**	CRF	Alfentanil delivered	DelALF	Alfentanil mg	continuous	Table 3	n, Median, min max, IQR	intra
**34.**	CRF	Propofol delivered	DelPROP	Propofol mg	continuous	Table 3	n, Median, min max, IQR	intra
**35.**	CRF	Rescue sedation	Resque_sed	0=no<br>1=yes	kategorisk/nominal	Table 3	P% x/n	intra
**36.**	CRF	Rescue reason	Rescue why	0=patient´s request<br>1=operator´s request<br>2=other	categorical/nominal	Table 3	P% x/n	intra

**37.**	CRF	Rescue type	Rescue type	0=propofol/ alfentanile<br>1=othert	categorical/nominal	Table 3		intra
**38.**	CRF	Hypoxia during procdure (Sp02<90 % or significant difference compared to baseline >5%SpO2)	hypoxia	0=no<br>1=yes	categorical/nominal	Table 3	P% x/n	intra
**39.**	CRF	Bradypnoea capnographic ≤8/min	RR	0=no<br>1=yes	categorical/nominal	Table 3	P% x/n	intra
**40.**	CRF	Chin llift	Chin_lift	0=no<br>1=yes	categorical/nominal	Table 3	P% x/n	intra
**41.**	CRF	Mask ventilation	Mask_vent	0=no<br>1=yes	categorical/nominal	Table 3	P% x/n	intra
**42.**	CRF	general anesthesia	anesth	0=no<br>1=yes	categorical/nominal	Table 3	P% x/n	intra
**43.**	CRF	Tacycardia >100	Takykard	0=no<br>1=yes	categorical/nominal	Table 3	P% x/n	intra
**44.**	CRF	Bradycardia <40	Bradykard	0=no<br>1=yes	categorical/nominal	Table 3	P% x/n	intra
**45.**	CRF	Bradycardia treatment	brady treat	0=atropine<br>1=efedrin	categorical/nominal	Table 3	P% x/n	intra
**46.**	CRF	Hypotension (SBP <90mmHg) or difference >30%	Low_SBP	0=no<br>1=yes	categorical/nominal	Table 3	P% x/n	intra
**47.**	CRF	Hypotension treatment	Hypo treat	0=vätska<br>1=sympatomimetic	categorical/nominal	Table 3	P% x/n	intra
**48.**	CRF	nausea	nausea	0=no<br>1=yes	categorical/nominal	Table 3	P% x/n	intra
**49.**	CRF	Patient back to post op time	Back_DUVA	Hh:mm	continuous	Table 3	n, Median, min max, IQR	post
**50.**	CRF	Patient leaving post op time	Disch_DUVA	Hh:mm	continuous	Table 3	n, Median, min max, IQR	post

**51.**	CRF	Postop time	Postop time	Leave postop (hh:min) – arrive postop (hh:min) efter procedure in minutes	continuous	Table 3	n, Median, min max, IQR	post U
**52.**	questionnaire	Overall satisfaction	Ov_satis	0-10	ordinal	Fig 1	n, Median, min max, IQR	post
**53.**	questionnaire	Satisfaction with staff	Staff_satis	0-10	ordinal	Fig 1	n, Median, min max, IQR	post
**54.**	questionnaire	Maximal pain(NRS) during procedure	NRS_max	0-10	ordinal	Fig 1	n, Median, min max, IQR	post
**55.**		1 no pain NRS 0-3<br>2 Moderate pain NRS 4-7<br>3 severe pain 8-10	NRS grupper	1-3	ordinal	Fig 1	n, Median, min max, IQR	post
**56.**	questionnaire	Maximal pain arm with infusion	Pain_arm	0-10	ordinal	Fig 1	n, Median, min max, IQR	post
**57.**	questionnaire	Satisfaction pain management	Pain_satis	0-10	ordinal	Fig 1	n, Median, min max, IQR	post
**58.**	questionnaire	Importance of sedation	Sed_import	0-10	ordinal	Fig 1	n, Median, min max, IQR	post
**59.**	questionnaire	Importance of control over sedation	Contr_import	0-10	ordinal	Fig 1	n, Median, min max, IQR	post
**60.**	QoR-15 post	15 items	QoR-15post	0-10	ordinal	Manus 2	n, Median, interkvartil	QoR-15 post
**61.**	QoR-15 tel	15 items	QoR-15 tel	0-10	ordinal	Manus 2	n, Median, interkvartil	QoR-15 tel

6 General Considerations

6.1 Data requisition

Descriptive patient data is supplied by the patient´s journal Cosmic or the National Patient Overview (NPÖ).

Procedurerelated data, medical parameters, registration of sedationrelated events is supplied by the patient´s anaesthesia journal. Trialspecific documentation is performed in electronic case report form (REDCap) and in the patient´s anaesthesia journal.

Patient´s self-reported intraprocedural pain assessment and satisfaction is registered on a patient inquiry. The inquiry is completed by the patient prior to discharge from the recovery unit.

6.2 Timing of Analyses

The final analysis will be performed after 340 patients are included in the trial. The trial ends with the last patient being the last subject of the trial.

The final analysis will be performed on data transferred to the file “database PACSPI 2”, having been documented as meeting the cleaning and approval requirements of the Trial Steering Group (TSG).

6.3 Analysis Populations

6.3.1 Full Analysis Population

All participants who were randomized to either group satisfying the exclusion criteria.

6.3.2 Per Protocol Population

All participants who received a SVP and completed the patient questionnaire.

6.3.3 Intention to Treat Analysis

Analyses will be performed on all participants in the study and control arms respectively which they were randomized to following the intention-to-treat principle.

6.4 Subgroups

Subgroup analyses are planned on centre, gender, operator´s experience, age, and cancer treatment strategy.

6.5 Missing Data

Missing data will be quantified and reported in the final manuscript by ”common-point imputation”.

6.6 Interim Analyses and Data Monitoring

6.6.1 Interim Analyses and Data Quality

Interim analyses are not expected to lead to early closure of neither randomization on safety nor efficacy grounds. Both techniques are established and clinically approved methods and complications are very uncommon. After 100 respectively 200 patients analyses of adverse events (AE) will be performed in which we expect a frequency lower than 15% based on previous studies [10]. In the event of a higher frequency the sponsor will discuss further action with the monitor (futility analysis might be considered).

Data validation will be performed after 5 and 10 patients were included in the trial to control REDCap´s functionality.

All staff involved in the trial will be informed on aim, progression, design and everything that lies in the staff´s responsibility according to profession.
Primary data sources are defined as patient journal, anaesthesia journal, e-CRF and patient inquiry and access will be granted to the monitor and controlling institutions on request. The required informed consent is included in the patient information/consent form.

6.6.2 Stopping Rules

No stopping rules apply.

6.6.3 Bias

Due to the nature and effects of sedative medication study participation is unblinded for both staff and participants. All patients scheduled for SVP will be screened for inclusion into the trial. The final analysis is performed by the chief investigator and statistician who remain blinded due to assessor blinding and coding in randomized groups.

6.7 Multi-centre Studies

This trial will be carried out in the following anaesthesia departments:
OP/IVA-klinken, County Hospital Ryhov, Sweden
AnOpIVA-kliniken, University Hospital Linköping, Sweden

Co-investigators at participating sites will have access to the REDCap. Centres are provided with the PCS pump. There will be no restrictions in the choice of LA-solution being left up to local practice at participating sites

6.8 Multiple Testing

Not applicable

7 Summary of Study Data

7.1 Data collection

All patients will be registered at OP/IVA department, County Hospital Ryhov,Jönköping, Sweden. For all data being collected see list of variables (section 5.5).

The following data will be recorded:

a) Demographic data: including patient´s age, gender, date of birth, length, weight, ASA-classification
b) Tumour presence: chemotherapy strategy
c) Device details: manufacturer, type, previous long-time access
d) Procedure details: implanting conditions, operator status, type and amount of local anaesthetic, access vein, number of needle passes, line tip position, use of ultrasound,
e) Vital parameters: heart rate, blood pressure, oxygen saturation at baseline
f) Sedation assessment: depth of sedation at four time points, administered amount of sedation/analgesic agents, need for rescue sedation and reason
g) Complication data: procedural complications, vital parameters complications due to sedation, unexpected airway management
h) Periprocedural time consumption
i) Health economic aspects
j) Patient´s pain perception and satisfaction: questionnaire before discharge
k) QoR-15 : at 3 timepoints, pre- and postoperatively and one day after discharge

7.2 Derived variables

Time variables will be derived into time consumption. BMI will be derived from patients´ length and weight.

7.3 Electronic case report form (e-CRF)

REDCap will be supplied by University of Linköping, Sweden. These e-forms are to be completed in accordance with the CRF completion guidelines issued for the study. All e-CRFs will be accessible to the chief investigator. E-CRFs will be stored in line with current regulatory requirements, that is, until 10 years after completion of the study. Essential documents such as source data and consent forms will be archived in line with current regulatory requirements and made available for monitoring and regulatory inspection.

7.4 Follow-Up and End of Trial

Patients will be followed up until completion of QoR-15 day 1 after discharge. The end of trial will be defined as last patient last discharge.

8 Study Management

8.1 Trial Steering Group (TSG)

The TSG will oversee the running of the trial. Members of the TSG will include the Chief Investigator and Co-Investigators.

8.2 Data and Safety Monitoring Board (DSMB)

An independent data and safety monitoring board (DSMB) will be established for the trial (FORUM, Linköping). The members will have a meeting with the chief investigator prior to study commencement to discuss the protocol as well as content and format of the DSMB reports.
The DSMB will once during the trial and at the end of the trial assess the progress of the trial, the safety data and efficacy endpoints according to the monitoring plan.

8.3 Annual Development safety Update Report, (DSUR)

As long as the trial progresses annual reports will be send to the Swedish Ethical Review Authority and Swedish Medical Products Agency. The report will state the applicable trial time period, a list of all SAE and SUSAR and contain assessment of safety and risk.

8.4 Ethical considerations

Ethical approval was sought from the Swedish Ethical Review Authority (Dnr 2022-00088-01). Amendment was granted October 2022 (Dnr 2022-00088-02) Written informed consent will be obtained from all participants in the study. This trial will be conducted in compliance with all stipulations of this protocol, the conditions of the Swedish Ethical Review Authority, the conditions in the Swedish Medical Products Agency, standards of Good Clinical Practice (defined by the International Conference on Harmonisation), ethical principles that have their origin in the Declaration of Helsinki and all applicable national and local regulations. This study is not sponsored by any commercial entity but was funded by Futurum (Academy for Healthcare, Jönköping County, Sweden, grant number: Futurum-963747). The trial will adhere to the Consolidated Standards of Reporting Trials (CONSORT) guidelines and is registered at EudraCT. (EudraCT Nr: 2021-003821-31).

9 Swedish Medical Products Agency and European Medical Agency

The trial falls under the requirements of the Medicinal Products for Human Use (Clinical Trials) Regulation. Registration at the European Union Drug Regulating Authorities Clinical Trials Database (EudraCT) has been performed and approval from the Swedish Medical Products Agency was sought and granted. EudraCT number 2021-003821-31.

10 Adverse Events

10.1 Definition AE, SAE and SUSAR

AE: An adverse event (AE) is any untoward medical occurrence in a patient administered a pharmaceutical product and which does not necessarily have a causal relationship with this treatment. An AE can therefore be any unfavourable and unintended sign, symptom, or disease temporally associated with the use of a medicinal product, whether or not related to the medicinal product. All AE are documented on a separate form. Adverse events not separately documented are listed in Table 1.

Table 1

Symptom	Definition	Intervention
Respiratory depression	Bradypnea: breaths <8/min	verbal stimulation and advice to use PCS less frequent<br>control patent airway<br>chin lift/jaw thrust,<br>establish patent airway oro-or naso-pharyngeal<br>bag-valve mask ventilation
Desaturation	Saturation <90 %	Increase oxygen flow<br>advice to use PCS less frequent<br>check airway, proceed as under respiratory depression if needed
Compromised airway	1. patent<br>2. snoring<br>3. obstructed	If 2: advice patient to use PCS less frequent, chin lift.<br>If 3 advice patient to use PCS less frequent, chin lift/jaw thrust;

		oro-or naso-pharyngeal airway; bag-valve mask ventilation<br><br>Laryngeal mask or intubation
Bradycardia	Heart rate <40 / min	Atropin<br><br>Efedrin
Hypotension	Hypotension (SBP <90mmHg) or difference >30% compared to baseline	Efedrin<br><br>Fluid bolus<br><br>Noradrenaline

SAE: A serious adverse event (SAE) is defined as any untoward medical occurrence that at any dose results in death, is life-threatening, requires inpatient hospitalization or causes prolongation of existing hospitalization, results in persistent or significant disability/incapacity, or requires intervention to prevent permanent impairment or damage. The investigator will follow all AE and SAE until judged to be clinically non-significant. Action taken and outcome of the AE/SAE are reported in the AE/SAE form.
All SAE are reported on a specific form to the sponsor within 24 hours.

SUSAR: A SUSAR (suspected unexpected serious adverse reaction) will be reported to the Swedish Medical Product Agency and Ethics committee according to the following time frame. "Unexpected" means that for an authorised medicinal product that the event is not described in the product's SmPC. In the case of SUSAR we will seek the Swedish Medical Product Agency´s help with reporting using CIOMS form due to the non-commercial nature of this trial. SUSAR resulting in death or lift-threating condition are reported within 7 days after the sponsor has been informed. All other SUSAR are reported within 15 days of the incident.

10.2 Causality and severity of AE and SAE

The principal investigator is responsible for judgement of causality and severity of reported AE/SAE and application of used medicinal products in this trial. Reporting is carried out on AE/SAE forms and the incident will be described and graded with date, causality, severity and result. Follow-up of patients with signs of AE/SAE is granted until the condition has subsided.

Causality: All AE are categorized according to the following definitions.
Probable causal relationship:
Evidence to suggest a causal relationship; the influence of other factors is unlikely.
Possible causal relationship:

Some evidence to suggest a causal relationship (e.g. occurrence within a reasonable time after administration of the trial medication), but other factors may have contributed to the event (e.g. another clinical condition or other concomitant treatment).
Unrelated causality:
No evidence of any causal relationship with the trial intervention.

Severity: All AE are categorized according to the following definitions.
Mild: AE results in awareness of sign or symptom, but easily tolerated
Moderate: AE results in discomfort sufficient to cause interference with normal activities
Severe: AE results in incapacitating, with inability to perform normal activities

11 Statistics and Data Analysis

Data will be summarised by treatment group. Number (n), median, interquartile range, minimum and maximum will summarise continuous efficacy variables, whereas number and percent will summarise categorical efficacy variables.

All analyses of the continuous efficacy variables will be performed as analysis of variance with the treatment group.

All summary tables will be structured with a column for each treatment and will be annotated with the total population size relevant to that table/treatment, including any missing observations.

Mann-Whitney U and Chi^2 will be used to test for comparison between groups, depending on whether data are discrete or continuous.

Primary objective: PCS with propofol and alfentanil as adjunct to LA reduces pain compared to LA only in SVP-implantation.

The minimum requirement is to demonstrate that the addition of PCS to LA reduces pain perception compared to SVP-implantation with LA only. Based on the assumption that 25% of patients experience pain ≥4 on NRS scale under SVP-implantation, we aim to detect at least a 50% reduction with PCS as adjunct. To detect this reduction with 80% power, 2-sided, significance level 5% requires 308 patients in total using 1:1 randomization. With an assumed drop-out rate of 10% we aim to include 170 patients per arm to show a difference between study arms.

11.1 Subject Disposition

Screening log will be used to document screening and subsequent enrolment of patients.

11.2 Protocol Deviations

Protocol deviations will be reported. Significant changes in the protocol will only be made after approval of the EPM and the LMV. Information on trial completion will be send to the

aforementioned authorities within 90 days after completion. Report in EudraCT´s database will be made within 12 months after trial completion.
Significant deviations comprise the following:
- changes that may compromise the patient´s safety or their physical and psychological wellbeing.
- changes that compromise the scientific nature of the trial
- changes that are significant of any other reason
Changes affecting a new trial site, new co-investigator or new patient consent form needs approval of the EPM.

11.3 Demographic and Baseline Variables

The summary statistics will be produced in accordance with section 11.

11.4 Prior and Concurrent Medications

Anaesthesiological assessment includes evaluation of regular medication.

11.5 Treatment Compliance

Not applicable

11.6 Pregnancies

Pregnant women are not enrolled.

12 Patient satisfaction data

Lack of a validated intervention-specific patient-related experience measure (PREM) questionnaires mandates use of a trial-specific questionnaire. This questionnaire addresses intervention-specific quality issues (pain, satisfaction with procedure) and interpersonal issues (satisfaction with the staff) on an 11-point numeric rating scale (NRS). The questionnaire is completed postprocedurally before discharge home.

13 Patient perioperative experience and recovery

Quality of recovery-15 (QoR-15) is a validated psychometric patient-centred outcome measure after day case surgery. The QoR-15 questionnaire will be answered by the patient at 3 timepoints. Preoperative, postoperative before discharge home and by telephone on day 1 after the procedure.

14 Confidentiality

Participants´ identification data will be required for the registration process. Preservation of confidentiality of participants is mandatory and registered under the data protection act.

15 Figures

Baseline variables, procedural characteristics and sedation variables will be communicated in tabulation. Primary and secondary endpoints will be communicated as table or figure according to section 17.

Flow sheet log Fig 1

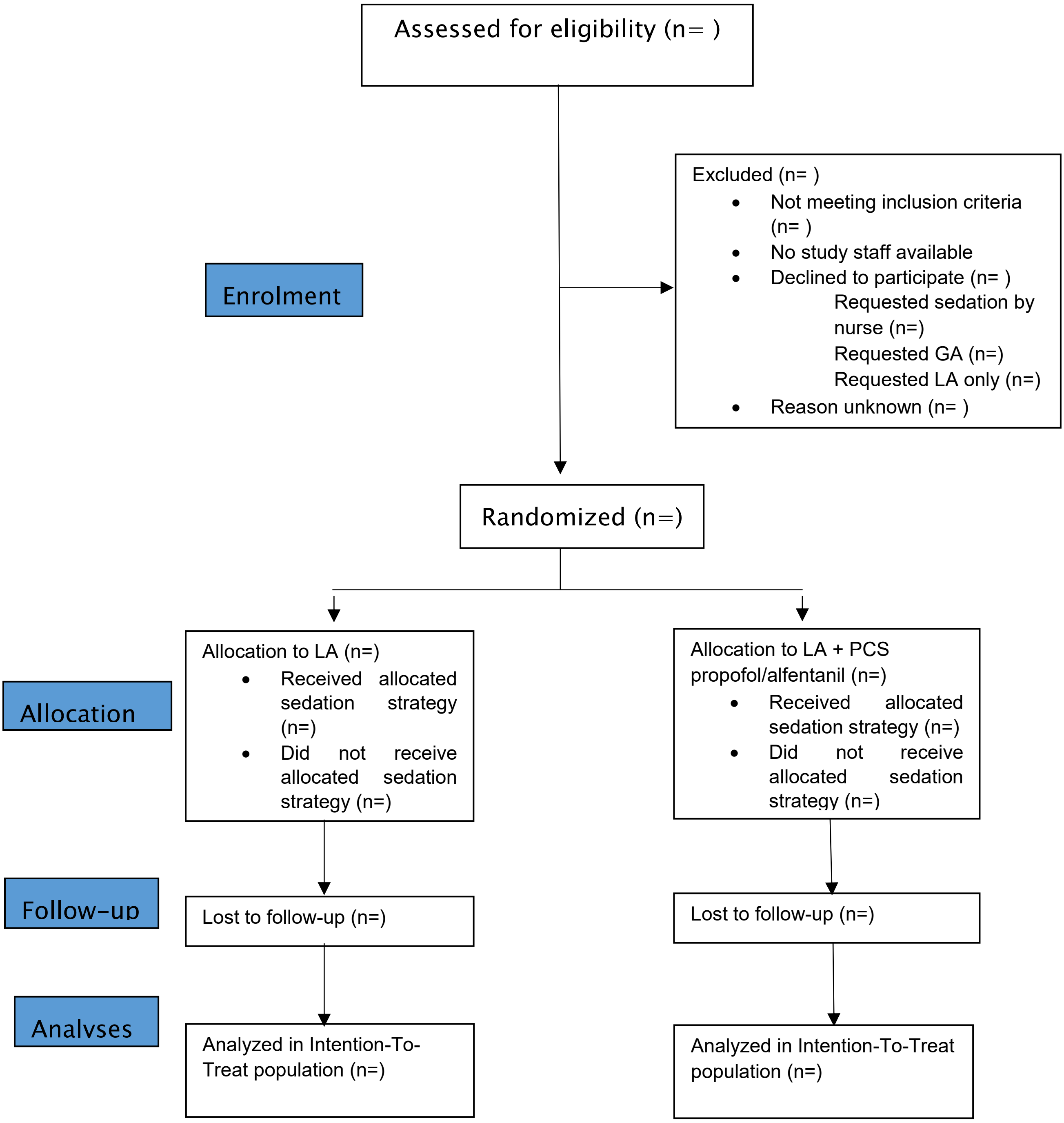

16 Reporting Conventions

P-values ≥ 0.01 will be reported to two decimal places, p-values ≥0.001 will be reported to three decimal places; p-values less than 0.001 will be reported as "<0.001". The median and any other statistics other than quantiles, will be reported to one decimal place greater than the original data. Quantiles, such as median, or minimum and maximum will use the same number of decimal places as the original data. Estimated parameters, not on the same scale as raw observations will be reported to 3 significant figures.

17 Funding

The study is funded by Futurum, Jönköping,Sweden grant number: Futurum-963747

18 Listing of Tables, Listings and Figures

Table 1 will display patient characteristics, containing variable 2-13.

Table 2 Patient characteristics. Data are presented as number (%) or median (minimum-maximum)

	LA	LA + PCS
Variable 2		
Variable 13		

Table 2 will display catheter and procedural characterististics, containing variable 14-30.

Table 3 Catheter and procedural characteristics and adverse events. Data are presented as number (%) or median (minimum-maximum)

	LA	LA + PCS
Variable 14		
Variable 30		

Table 3 will display sedation characteristics and adverse events, containing variable 31-

Table 3 Sedation characteristics and adverse events. Data are presented as number (%) or median (minimum-maximum)

	LA	LA + PCS
Variable 31		

Variable 51		

Figure 1 Patients´ self-reported perception and assessment. Data are presented as number (%) or median (minimum-maximum)

Figure 2 will contain patients´ self-reported perception and assessment. Variable 52-59 will be displayed in a chart.

QoR-15 data collected att 3 timepoints will be presented in a separate manuscript.

19 References

1. Årsrapport SPOR 2020. Swedish perioperative registry (2020); https://spor.se/spor/arsrapporter/. Accessed 26 November 2021
2. Taxbro K, Hammarskjöld F, Thelin B, Lewin F, Hagman H, Hanberger H, et al. (2019). Clinical impact of peripherally inserted central catheters vs implanted port catheters in patients with cancer: an open-label, randomised, two-centre trial. Br J Anaesth;122(6):734-41. http://dx.doi.org/10.1016/j.bja.2019.01.038.
3. Grossmann B, Nilsson A, Sjöberg F, Bernfort L, Nilsson L. (2020). Patient-controlled sedation with propofol for endoscopic procedures-A cost analysis. Acta Anaesthesiol Scand;64(1):53-62. http://dx.doi.org/10.1111/aas.13463.
4. Kreienbühl L, Elia N, Pfeil-Beun E, Walder B, Tramèr MR. (2018). Patient-Controlled Versus Clinician-Controlled Sedation With Propofol: Systematic Review and Meta-analysis With Trial Sequential Analyses. Anesth Analg;127(4):873-80. http://dx.doi.org/10.1213/ane.0000000000003361.
5. Grossmann B, Nilsson A, Sjöberg F, Nilsson L. (2020). Patient-controlled Sedation During Flexible Bronchoscopy: A Randomized Controlled Trial. Journal of bronchology & interventional pulmonology;27(2):77-85. http://dx.doi.org/10.1097/lbr.0000000000000610.
6. Clements W, Sneddon D, Kavnoudias H, Joseph T, Goh GS, Koukounaras J, et al. (2018). Randomized and controlled study comparing patient controlled and radiologist controlled intra-procedural conscious sedation, using midazolam and fentanyl, for patients undergoing insertion of a central venous line. J Med Imaging Radiat Oncol;62(6):781-8. http://dx.doi.org/10.1111/1754-9485.12817.
7. Burlacu CL, McKeating K, McShane AJ. (2011). Remifentanil for the insertion and removal of long-term central venous access during monitored anesthesia care. J Clin Anesth;23(4):286-91. http://dx.doi.org/10.1016/j.jclinane.2010.12.007.
8. Vardon Bounes F, Pichon X, Ducos G, Ruiz J, Samier C, Silva S, et al. (2019). Remifentanil for Procedural Sedation and Analgesia in Central Venous Catheter Insertion: A Randomized, Controlled Trial. Clin J Pain;35(8):691-5. http://dx.doi.org/10.1097/ajp.0000000000000725.
9. Chernik DA, Gillings D, Laine H, Hendler J, Silver JM, Davidson AB, et al. (1990). Validity and reliability of the Observer's Assessment of Alertness/Sedation Scale: study with intravenous midazolam. J Clin Psychopharmacol;10(4):244-51.
10. Preprint Researchsquare; Patient-Controlled Sedation in Port Implantation (PACSPI 1) – a Feasibility Trial. BMC Anesthesiology (2022); https://www.researchsquare.com/browse?unified=pacspi&offset=0&status=all. Accessed 2022-03-23

20 Supplements

Bilaga 1

Venportsinläggande läkares skattning av prodcedurkvalitet/operationsbetingelser

1. Proceduren kan genomföras utan påverkan i tid eller anpassning
2. Proceduren utförs med viss påverkan i tid eller anpassning
3. Anpassning krävs för procedur med god kvalitet, längre tidsåtgång
4. Extra läkemedel krävs alt proceduren kan inte utföras

Bilaga 2

OAA/S sederingsskala

Reaktion	Score
Reagerar snabbt på tilltal vid normalt röstläge	5
Slö reaktion på tilltal vid normalt röstläge	4
Reagerar endast på tilltal med höjt röstläge eller upprepade tilltal	3
Reagerar efter mild beröring/skakning	2
Reagerar på smärtstimulering	1
Ingen reaktion på smärtstimulering	0

Content	Score
Responds readily to name spoken in normal tone	5
Responds lethargically to name spoken in normal tone	4
Responds only after name is called loudly, repeatedly, or both	3
Responds only after mild prodding or shaking	2
Responds only after painful trapezius squeeze	1
Does not respond to painful trapezius squeeze	0

Sederingsskala i original

Bilaga 3

Patientenkät

1.	Hur nöjd är du med ditt omhändertagande i samband med venportsinläggnig?	Inte alls nöjd	1	2	3	4	5	6	7	8	9	10	Mycket nöjd
2.	Hur upplevde du kontakten med personalen du har träffat i samband med venportsinläggningen?	Inte alls bra	1	2	3	4	5	6	7	8	9	10	Mycket bra
3.	Hur mycket smärta hade du som mest i samband med venportsinläggningen?	Inte alls ont	1	2	3	4	5	6	7	8	9	10	Värsta smärtan jag vet
4.	Hur nöjd är du med din smärtbehandling under ingreppet?	Inte alls nöjd	1	2	3	4	5	6	7	8	9	10	Mycket nöjd
5.	Är det viktigt att få lugnande medicin under ingreppet?	Inte alls viktigt	1	2	3	4	5	6	7	8	9	10	Mycket viktigt
6.	Hur mycket smärta upplevde du i armen där infusionen var kopplad?	Inte alls ont	1	2	3	4	5	6	7	8	9	10	Värsta smärtan jag vet
7.	Hur viktigt är det för dig att själv kunna kontrollera tillförseln av lugnande medicin?	Inte alls viktigt	1	2	3	4	5	6	7	8	9	10	Mycket viktigt

QoR-15swe (Quality of Recovery-15 Sweden)

Del A
Hur har du mått de senaste 24 timmarna?

På en skala från 0 till 10, där 0 = Inte någon gång {dåligt} och 10 = hela tiden {utmärkt}

**1**	**Kunnat andas lugnt**	**Inte någon gång**	**0**	**1**	**2**	**3**	**4**	**5**	**6**	**7**	**8**	**9**	**10**	**hela tiden**
**2**	Kunnat njuta av maten	Inte någon gång	0	1	2	3	4	5	6	7	8	9	10	hela tiden
**3**	Känt dig utvilad	Inte någon gång	0	1	2	3	4	5	6	7	8	9	10	hela tiden
**4**	Kunnat sova gott	Inte någon gång	0	1	2	3	4	5	6	7	8	9	10	hela tiden
**5**	Kunnat sköta toalettbesök och personlig hygien utan hjälp	Inte någon gång	0	1	2	3	4	5	6	7	8	9	10	hela tiden
**6**	Kunnat kommunicera med anhöriga eller vänner	Inte någon gång	0	1	2	3	4	5	6	7	8	9	10	hela tiden
**7**	Fått stöd från sjukhuspersonal	Inte någon gång	0	1	2	3	4	5	6	7	8	9	10	hela tiden
**8**		Inte någon gång	0	1	2	3	4	5	6	7	8	9	10	hela tiden

	Kan du utföra ditt arbete eller dina vanliga aktiviteter hemma													
**9**	Känt dig trygg och haft kontroll över din tillvaro	Inte någon gång	0	1	2	3	4	5	6	7	8	9	10	hela tiden
**10**	Haft en känsla av allmänt välbefinnande	Inte någon gång	0	1	2	3	4	5	6	7	8	9	10	hela tiden

Del B
Har du känt något av följande symptom de senaste 24 timmarna?

på en skala från 10 till 0, där 10 = Inte någon gång {utmärkt} och 0 = hela tiden {dåligt}

**6**	**Medelsvår smärta**	**Inte någon gång**	**0**	**1**	**2**	**3**	**4**	**5**	**6**	**7**	**8**	**9**	**10**	**hela tiden**
**7**	Svår smärta	Inte någon gång	0	1	2	3	4	5	6	7	8	9	10	hela tiden
**8**	Illamående eller kräkning	Inte någon gång	0	1	2	3	4	5	6	7	8	9	10	hela tiden
**9**	Känt ångest eller oro	Inte någon gång	0	1	2	3	4	5	6	7	8	9	10	hela tiden
**10**	Känt dig ledsen eller deprimerad	Inte någon gång	0	1	2	3	4	5	6	7	8	9	10	hela tiden

Bilaga 4
SFAI fasteregler

Preoperativ fasta vuxna och barn.

Sammanfattning av riktlinjer för preoperativ fasta som publicerats av European society of Anaesthesiology 2011. (Smith I, Kranke P, Murat I, Smith A, O'Sullivan G, Søreide E, Spies C, in't Veld B; European Society of Anaesthesiology. Perioperative fasting in adults and children: guidelines from the European Society of Anaesthesiology. *Eur J Anaesthesiol*. **2011**; 28(8): 556-69)

Sammanfattning gjord av Karolina Persson ST läkare Lund och Gunilla Islander riktlinjesredaktör.
Accepterad av SFAIs styrelse 2013-03-12

Preoperativ fasta vuxna och barn.

Kortfattat: *Inför en anestesi kan/bör patienter dricka klara vätskor fram till två timmar och äta fram till sex timmar innan anestesistart.*

	Evidens	Rekomm-endation
**Fasta innan operation; vuxna och barn**		
Vuxna och barn skall uppmanas att dricka klara vätskor (vatten, juice utan fruktkött, kaffe eller te utan mjölk) fram till 2 timmar innan en planerad operation (inkl. kejsarsnitt)	1++	A
En majoritet av författarna godkände mjölk i kaffe eller te upptill 1/5 av volymen. Konsensus kunde ej nås.		√
Sex timmars preoperativ fasta för allt utom klara vätskor som definierats ovan.	1+	A
Patienter med fetma, gastroesofageal reflux, diabetes eller gravida kvinnor som inte är i förlossningsarbete kan följa dessa rutiner. *Notera att dessa tillstånd i sig kan påverka det anestesiologiska omhändertagandet.*	2-	D
Anestesier skall inte skjutas upp för att patienten tuggat tuggummi sugit på en karamell eller rökt en cigarett innan planerad anestesistart.	1-	B
Detta baseras enbart på effekten på ventrikeltömningen. Man skall avråda från allt nikotinintag; rökning, tuggummi eller plåster innan elektiv kirurgi.		√
**Fasta innan operation för spädbarn.**		
Spädbarn skall få näring innan elektiv kirurgi. Bröstmjölk kan ges fram till 4 timmar innan anestesi, övrig mjölk fram till 6 timmar. Därefter kan klara vätskor ges som till vuxna.	1++	A

Motorikstimulerande och annan farmakologisk intervention

Det finns inte tillräckliga bevis för att rekommendera rutinanvändning av antacida, metoclopramid, eller H2 receptorblockerare innan elektiv kirurgi hos icke-obstetriska patienter.

H2 receptorblockerare skall ges kvällen innan och på operationsdagens morgon inför ett elektivt kejsarsnitt. *Det skall noteras att detta råd baseras på surrogatmått såsom volym och pH av magsaft och inte på några studier av mödradödlighet.*	1++	A
En intravenös H2 receptorblockerare skall ges innan ett akut kejsarsnitt; Detta skall kompletteras med 30 ml av 0,3 mol l^{-1} natrium-citrat om generell anestesi planeras. *Det skall noteras att detta råd baseras på surrogatmått såsom volym och pH av magsaft och inte på några studier av mödradödlighet.*	1++	A

Kolhydrater per os

Det är utan risk för patienter (diabetiker inkluderade) att dricka kolhydratrik vätska fram till 2 timmar innan elektiv kirurgi. *Evidensen är baserad på studier av kolhydratinnehållande produkter som utvecklats speciellt för preoperativt bruk (vanligtvis maltodextriner); det innebär att det är inte säkert att alla kolhydratinnehållande vätskor är riskfria*	1++	A
Att inta koldhydratrika drycker innan elektiv kirurgi ökar det subjektiva välbefinnandet, minska törst och hunger och minskar postoperativ insulinresistens. *Det finns inga evidens för att längden på postoperativ vård eller mortalitet påverkas.*	1++	A

Fasta obstetriska patienter

Kvinnor i förlossningsarbete skall tillåtas att fritt dricka klara vätskor (definition se ovan).	1++	A
Under pågående förlossningsarbete skall kvinnan avrådas från att äta mat. *Författarna till riktlinjen inser att detta kan vara svårt att genomföra framför allt hos lågrisk patienter, Lättsmält slaggfri föda, bör has i åtanke.*	1+	A

När skall patienten börja dricka igen efter narkos/operation

Vuxna och barn skall tillåtas att dricka så snart de så önskar efter elektiv kirurgi. Patienten behöver inte nödvändigtvis ha druckit innan hemgång från poliklinisk narkos.	1++	A

2(3)

Förklaringar

Evidens

1++ Baserat på högkvalitativa metaanalyser, systematiska review-undersökningar av randomiserade kontrollerade studier (RCT) eller RCT med mycket liten risk för bias.

1+ Väl genomförda meta-analyser, systematiska review-undersökningar,

1- Meta-analyser, systematiska review-undersökningar eller RCT med stor risk för bias.

2++ Högkvalitativa systematiska case-control studier eller cohort studier med liten risk för confounding factors.

2+ Väl genomförda case control studier med liten risk för confounders eller bias och en moderat probabilitet att relationen är kausal.

2- Case-control studier eller cohort studier med hög risk för confounders eller bias och signifikant risk för att relationssambandet inte är kausalt.

3 Icke analytiska studier e.g. fallbeskrivningar eller fallserier.'

4. Expert opinion

Gradering av rekommendationerna

Graderingen av rekommendationerna baseras på styrkan av evidensen. Det reflekterar inte klinisk betydelse.

A Minst en meta-analys, systematisk review eller RCT klassad som 1++.
och direkt applicerbar på målpopulationen eller
flera studier som ger evidens som huvudsakligen är klassade som 1+ och
direkt applicerbar på målpopulationen eller visar en generell samstämmighet av resultaten.

B Flera studier inkluderande studier som klassats som 2++ och som är direkt applicerbara på målpopulationen samt visar samstämighet i resultaten eller extrapolerade evidens från studier som klassats som 1++ eller 1+.

C Flera studier inkluderande studier som klassats som 2+ och som är direkt applicerbara på målpopulationen samt visar samstämighet i resultaten eller extrapolerade evidens från studier som klassats som 2++.

D Evidensklass 3 eller 4
Extrapolerade evidens från studier som klassats som 2+.

√ Rekommenderad "best practice" baserat på klinisk erfarenhet hos författarna till denna riktlinje
